# Relationship between placental expression of the imprinted *PHLDA2* gene, intrauterine skeletal growth and childhood bone mass^☆^

**DOI:** 10.1016/j.bone.2011.11.003

**Published:** 2012-01

**Authors:** R.M. Lewis, J.K. Cleal, G. Ntani, S.R. Crozier, P.A. Mahon, S.M. Robinson, N.C. Harvey, C. Cooper, H.M. Inskip, K.M. Godfrey, M.A. Hanson, The Southampton Women's Survey Study Group, R.M. John

**Affiliations:** a Institute of Developmental Sciences, University of Southampton and Southampton University Hospitals NHS Trust, UK; b MRC Lifecourse Epidemiology Unit, University of Southampton and Southampton University Hospitals NHS Trust, UK; c Southampton NIHR Biomedical Research Unit in Nutrition, Diet & Lifestyle, University of Southampton and Southampton University Hospitals NHS Trust, UK; d Cardiff School of Biosciences, University of Cardiff, UK

**Keywords:** *PHLDA2*, Fetal growth, Bone mineral content, Imprinted genes, Placenta

## Abstract

Alterations in expression of the imprinted gene *PHLDA2* are linked to low birth weight in both humans and the mouse. However birth weight is a summary measure of fetal growth and provides little information on the growth rate of the fetus in early and late pregnancy. To examine the relation of *PHLDA2* expression with rates of fetal growth and explore associations with the infant's body composition in early childhood, we measured *PHLDA2* mRNA levels in the term placenta of 102 infants whose mothers were participating in the Southampton Women's Survey (SWS). Higher *PHLDA2* expression was associated with a lower fetal femur growth velocity between 19 and 34 weeks gestation. In addition, higher placental *PHLDA2* gene expression was associated with a lower child's bone mineral content at four years of age, measured using dual-energy X-ray absorptiometry. The results suggest that placental *PHLDA2* may provide a biomarker for suboptimal skeletal growth in pregnancies uncomplicated by overt fetal growth restriction.

## Highlights

► We examined placental *PHLDA2* expression in 102 pregnancies. ► Identified negative correlation between placental *PHLDA2* and fetal femur growth. ► Identified negative correlation between placental *PHLDA2* and bone density at 4 years. ► Placental *PHLDA2* may provide a biomarker for suboptimal skeletal growth.

## Introduction

The most common cause of low birth weight (LBW) at term in Western societies is placental insufficiency [1]. LBW infants have not attained their growth potential (reviewed in [2]) but in addition birth weight is an important indicator of both short and long term health. LBW infants have a 10–20 fold increased risk of dying in the perinatal period [3] and are at increased risk of developing chronic diseases including type 2 diabetes, hypertension and heart disease in later life (discussed in [4,5]). Postnatal ‘catch up’ growth is observed in 70–90% of LBW infants and is generally complete by two years of age [6,7]. It is now widely accepted that human fetuses are able to respond to a limited supply of nutrients by changing their physiology and metabolism but this predisposes to chronic disease in later life [8]. There is now extensive data from animal models to support this hypothesis [9–14].

Elevated expression of the human *PHLDA2* gene has been reported in the term placentas of LBW infants in two independent studies [15,16]. In a study of routine, ultrasound-dated pregnancies, higher placental *PHLDA2* expression was also shown to correlate with lower birth weight [17]. *PHLDA2* belongs to a family of imprinted genes expressed from only the maternally-inherited allele [18]. In humans, *PHLDA2* is expressed primarily in the placenta in the villous cytotrophoblast until term [19]. Similarly, expression in the mouse is predominantly placental [18,20,21]. In mice *PHLDA2* gene knockout results in placentomegaly with an expansion of the junctional zone but has no apparent consequence for fetal weight, fetal viability or adult health [22]. In contrast, mice genetically engineered to over-express *PHLDA2* show a reduced placental weight and there is reduced fetal growth late in gestation, suggesting placental insufficiency [23,24]. Data from the mouse model suggest that excess expression of *PHLDA2* in the placenta of human LBW infants is not merely a consequence of a dysfunctional placenta but contributes to the reduction in growth. Determining placental *PHLDA2* expression may therefore help identify infants who have not reached their full growth potential. Distinguishing these infants may allow targeted interventions early in life to optimize adult health.

In this study, we examined *PHLDA2* expression in placentas of 102 infants born to mothers participating in the Southampton Women's Survey for whom there is detailed information about fetal growth and placental weight at term [25]. In addition to measurements of fetal growth velocity, we correlated placental expression of *PHLDA2* with the infant's anthropometry, bone mass and body composition at birth (measured by dual-energy X-ray absorptiometry (DXA)) and, where data were available, bone mass and body composition in early childhood. We found no significant relationship between *PHLDA2* expression and birth weight in this cohort, but there were relationships between higher placental *PHLDA2* expression and lower femur growth rate between 19 and 34 weeks of gestation and lower bone mineral content at 4 years.

## Subjects and methods

### Cohort

Details of the Southampton Women's Survey (SWS) have been published previously [25]. In a group of pregnancies the placenta was collected within 30 min of delivery. The weight of the placenta was measured after removing any obvious blood clots, cutting the umbilical cord flush with its insertion into the placenta, trimming away surrounding membranes and removing the amnion from the basal plate. To ensure that samples collected were representative of the placenta as a whole, 5 villous tissue samples were selected using a stratified random sampling method and stored at − 80 °C. For this study, we selected 102 placentas (from 300 collected in total) based on availability of neonatal DXA data.

### Fetal ultrasonography

In 58 pregnancies, measures of fetal size and growth velocity were available from ultrasound scans performed by a research sonographer at 19 and 34 weeks gestation. Using a high resolution ultrasound system (Kretz Voluson 730), head circumference (HC) was obtained using an ellipse superimposed on a static scan image of the horizontal plane at the level of the thalamus and the cavum septi pellucidi [26]. Abdominal circumference (AC) was also similarly measured using a transverse section of the fetal abdomen at the level of the fetal stomach and where a short section of umbilical vein can be identified. Femur length (FL) was measured in longitudinal section by placing the linear calipers at the ends of the diaphysis, with the femur horizontally positioned in the scan plane [26]. Three measurements were made of each parameter and the mean used in the statistical analysis [27]. Precision of the measurements was assessed by replicate examinations in 50 pregnancies at both 19 and 34 weeks. The coefficient of variation for triplicate linear measurements was 0.6% at 19 weeks and 0.4% at 34 weeks [27]. For elliptical measurements the values were 4.4% at 19 and 3.2% at 34 weeks.

### Postnatal measurements

The infant's gestational age at birth was calculated from the date of the mother's last menstrual period (LMP) and confirmed by ultrasonography, or adjusted by an early dating scan if the LMP was unsure. Shortly after delivery, research midwives recorded neonatal anthropometric measures (including birth weight, head, abdominal and mid-upper arm circumferences and crown–heel length).

A subset of 42 mothers and children were invited to visit the Osteoporosis Centre at Southampton General Hospital for assessment of bone mass when the child was 4 years old. At this visit written informed consent for the DXA scan was obtained from the mother or father. The child's height (using a Leicester height measurer) and weight (in underpants only, using calibrated digital scales (Seca Ltd.)) were measured. A whole body DXA scan was obtained, using a Hologic Discovery instrument (Hologic Inc., Bedford, MA, USA). To encourage compliance, a sheet with appropriate colored cartoons was laid on the couch first; to help reduce movement artifact, the children were shown a suitable DVD cartoon. The total radiation dose for the scans was 4.7 microsieverts for whole body measurement (pediatric scan mode). The manufacturer's coefficient of variation (CV) for the instrument was 0.75% for whole body bone mineral density, and the experimental CV when a spine phantom was repeatedly scanned in the same position 16 times was 0.68%.

### RNA extraction and cDNA synthesis

For each placenta 5 snap frozen samples were pooled and powdered in a frozen tissue press. Total RNA was extracted from 30 mg powdered placental tissue using the RNeasy fibrous tissue RNA isolation mini kit (Qiagen, UK) according to the manufacturer's instructions. The integrity of total RNA was confirmed by visualization of ribosomal bands with ethidium bromide under ultra violet illumination by agarose gel electrophoresis, in 1 × TAE buffer.

Total RNA (0.2 μg) was reverse transcribed with 0.5 μg random hexamer primer, 200 units M-MLV reverse transcriptase, 25 units recombinant RNasin ribonuclease inhibitor and 0.5 mM each of dATP, dCTP, dGTP and dTTP in a final reaction volume of 25 μl in 1 × MMLV reaction buffer (Promega, Wisconsin, USA). All 102 samples were produced in one batch to reduce variation.

### Probe and primer design

Oligonucleotide probes and primers were designed using the Roche ProbeFinder version 2.45 for human. Probes were supplied by Roche from the human universal probe library and primers were synthesized by Eurogentec (Seraing, Belgium). *PHLDA2*: Forward 5′-atcacttggccagtttgctt-3′, Reverse 5′-gactggatgagggtgtcctg-3′, probe #3. Control genes (*YWHAZ*, *UBC* and *TOP1*) were selected using the geNormTM human Housekeeping Gene Selection Kit (Primer Design Limited, Southampton UK).

Real-time PCR using a Roche light-cycler 480. For Roche universal probe library probes the cycle parameters were 95 °C for 10 min, followed by 40 cycles of 95 °C for 15 s and 60 °C for 1 min. For primer design Perfect Probes the cycle parameters were 95 °C for 10 min, followed by 40 cycles of 95 °C for 10 s and 60 °C and 72 °C for 15 s. Intra-assay CV's for each gene were 5–8%. Each of the 102 samples was run on the same plate in triplicate. All mRNA levels are presented relative to the geometric mean of the three control genes.

*PHLDA2* expression levels were quantified by Real-time PCR (QPCR) against three reference genes: *tyrosine 3-monooxygenase/tryptophan 5-monooxygenase activation protein, zeta polypeptide* (*YWHAZ*), *ubiquitin C* (*UBC*) and *topoisomerase* (*TOP1*) [28].

### Statistics

Summary data are presented as mean (SD) or median (inter-quartile range) depending on whether or not the data were normally distributed. Variables not normally distributed were transformed logarithmically. To investigate associations between *PHLDA2* expression and parental body composition, fetal growth rates and infants body composition, Pearson's and Spearman's correlation coefficients were calculated where appropriate. Differences in *PHLDA2* expression levels between different categories of maternal lifestyle were tested by *t-test* or one-way analysis of variance. Neonatal anthropometric measurements were adjusted for sex and gestational age and neonatal DXA measurements were adjusted for sex, gestational age and age at DXA. As there was a question regarding sex differences in mRNA levels between male and female placentas all mRNA data were adjusted for the sex of the baby [29]. Within group Z-scores were generated for femur length and abdominal circumference at 19 and 34 weeks. Royston models were fitted to fetal measurements to create z-scores for size and conditional growth rates [30]. To investigate whether there were sex differences in the relationship between *PHLDA2* expression and the variables sex was included in regression analyses as appropriate and where an interaction was found data were analyzed separately by sex. Data were analyzed using Stata version 11.0 (Statacorp, Texas, USA).

## Results

### Descriptive statistics

In this study, *PHLDA2* gene expression was examined in the placentas from 102 infants collected as part of the Southampton Women's Survey. All were singleton, term deliveries (37 weeks gestation or greater). 53 of the infants were male and 49 were female. Descriptive statistics are given in Table 1.

### Relationships between neonatal parameters and term placental expression of *PHLDA2*

Within this cohort of 102 infants, no association was found between the placental expression level of *PHLDA2* and birth weight, placental weight or other neonatal anthropometric or body composition measurements at birth (Table 2).

### Relationships between fetal growth parameters and term placental expression of *PHLDA2*

**Fig. 1:**
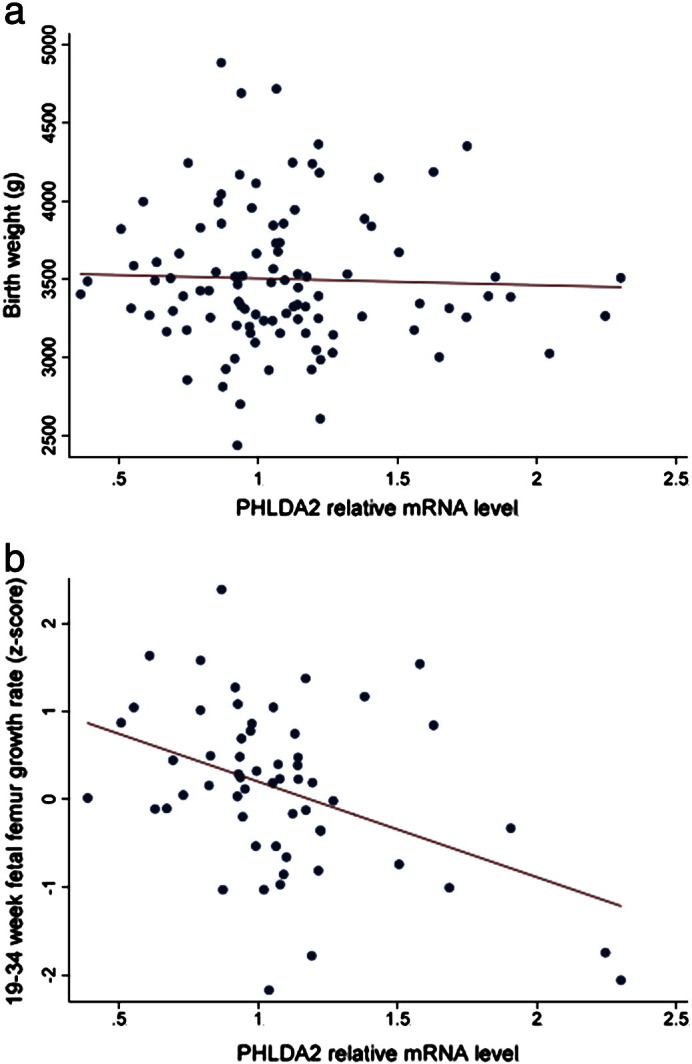
Placental *PHLDA2* relative mRNA levels at birth compared to a) birth weight (r = − 0.02, P = 0.88) and b) 19–34 week fetal femur growth velocity (r = − 0.42, P = 0.001) (Royston femur length Z score) [30].

Longitudinal fetal ultrasound data was available at both 19 and 34 weeks for 58 fetuses within the cohort of 102 infants. There were no differences in the birth parameters between this subset of 58 pregnancies and the 43 pregnancies without full fetal scan data (data not shown). A lower 19–34 week femur length z-score change (linear growth velocity) was significantly associated with higher term placental *PHLDA2* mRNA levels (Table 3, Fig. 1). Fetal femur length was not significantly related to placental *PHLDA2* expression at 19 weeks of gestation but shorter fetal femur length at 34 weeks was associated with higher term placental expression of *PHLDA2* (r = 0.35, P = 0.01) (Supplementary figure). This data suggests reduced femur growth velocity late in gestation in infants with a higher expression level of placental *PHLDA2*.

Placental expression of *PHLDA2* was not related to fetal head circumference z-score at 19 weeks or 19–34 week fetal head circumference growth velocity (Table 3). Fetal abdominal circumference z-scores at 19 were not significantly related to placental expression of *PHLDA2*, but higher *PHLDA2* expression was associated with a faster fetal abdominal circumference growth velocity between 19 and 34 weeks (Table 3).

### Relationships between placental expression of *PHLDA2* and childhood body composition at age 4 years

42 children in the study had DXA scans at age 4 years. There were no significant differences in the birth parameters collected for those who had DXA and those who did not (data not shown). In the 22 male and 20 female children followed to 4 years, there was an inverse relationship between placental *PHLDA2* gene expression and bone mineral content, bone area and bone mineral density determined by DXA (Table 4). There were no significant correlations between either bone lean mass or fat mass and *PHLDA2* expression when the data was analyzed by the sex of the infant or independently of sex (Table 4).

There were no significant interactions between *PHLDA2* mRNA levels and sex with any fetal, neonatal or postnatal outcomes.

### Parental parameters

Term placental *PHLDA2* mRNA levels were not associated with maternal parity primiparous vs multiparous (values are mean (SD), 1.1 (0.4) vs 1.0 (0.4), P = 0.21), smoking (non-smoking 1.1 (0.3) vs smoking 1.2 (0.6), P = 0.18) or social class (social class I/II1.0 (0.3), IIIN/M1.2 (0.4), IV/V1.0 (0.3) P = 0.30) but levels were higher in mothers who, at recruitment to the study, reported that they undertook strenuous exercise compared to those who did not (1.2 (0.4) vs 1.0 (0.3). P = 0.02).

Term placental *PHLDA2* mRNA levels were not associated with mother's own birth weight (r = 0.08, P = 0.47), height (r = − 0.05, P = 0.60), BMI (r = − 0.16, P = 0.11) or arm muscle area (r = − 0.10, P = 0.33). There were no significant interactions between term placenta *PHLDA2* mRNA levels and sex for any maternal anthropometric outcomes. A lower paternal birth weight was associated with higher term placental *PHLDA2* mRNA levels (R = − 0.35, P = 0.02).

## Discussion

Our first key finding was the negative correlation between linear femur growth rate between 19 and 34 weeks of gestation and *PHLDA2* expression in the term placenta in both male and female infants. A second finding was the negative correlation between bone mineral content at age 4 years and *PHLDA2* expression in the term placenta.

Two independent studies have reported increased placental *PHLDA2* expression at term in growth restricted infants [15,16]. In a third study, Apostolidou et al., reported a negative correlation between birth weight and placental *PHLDA2* in 200 routine pregnancies [17]. We did not identify a significant negative trend between *PHLDA2* expression in our 102 routine pregnancies which may suggest differences in the populations under study, differences in the way in which these measurements were taken or differences in the methodologies, for instance in our study we normalized to the geometric mean of three housekeeping genes which had been shown to be stably expressed in human placenta [28]. However, despite the lack of correlation with birth weight, we did identify a statistically significant negative correlation between *PHLDA2* expression and linear growth rate in the 58 infants who underwent ultrasound scanning at 19 and 34 weeks. There was also a weak negative correlation between placental *PHLDA2* and crown heel length at birth and also height at age 4, both of which might be anticipated to have a relationship with pre term femoral length. While these measurements did not reach significance, DXA data obtained at 4 years did reveal an inverse correlation between placental *PHLDA2* expression and bone mineral content [31]. These data suggest that *PHLDA2* may act to restrict skeletal growth and this restricted growth in utero has post natal consequences for skeletal integrity. As with birth weights, the lack of significant negative correlation of *PHLDA2* expression with crown heel length at birth and height at age 4 might be explained by the imprecision of measurements taken on a single occasion over that obtained from scan data. It may well be that with a larger cohort these negative trends will achieve significance.

We have previously reported a direct causative effect between high *PHLDA2* and late onset growth restriction in an animal model, which we attributed to placental insufficiency [23,24]. Data from an animal model employing bilateral uterine vessel ligation to mimic placental insufficiency suggests a link between suboptimal fetal growth and postnatal bone density [32]. *PHLDA2* may act specifically to limit the transport of factors required for skeletal growth, for example by limiting calcium transport. Alternatively, *PHLDA2* may indirectly affect calcium and bone metabolism through its role in regulating the placental hormones [24] involved in driving the maternal adaptations to pregnancy, which include increased maternal bone turnover. A third possibility is that *PHLDA2* acts intrinsically to limit bone growth. *PHLDA2* is expressed in human chondrocytes [33] and high expression of *PHLDA2* has recently been reported in hypertrophic mouse chondrocytes, as compared to proliferative/resting chondrocytes [34]. Animal models will play an important role in distinguishing between an intrinsic or extrinsic mechanism for limiting skeletal growth. Whatever the mechanism turns out to be, we have identified a potential role for *PHLDA2* in restricting early skeletal growth, which has post natal consequences for skeletal integrity.

In contrast to the negative relationship with fetal femur growth, *PHLDA2* was positively associated with change in abdominal circumference from 19 to 34 weeks. There is much evidence to suggest that different fetal compartments may be differentially regulated and thus growth may not be concordant across these measurements. Femur length may be taken as a proxy for linear growth of the skeleton (crown rump or crown heel length are not measurable by ultrasound in late pregnancy); in contrast abdominal circumference is a composite measure of liver size and thickness of subcutaneous adipose tissue, potentially involving hormones such as IGF-1 and leptin [35,36]. There is no reason to suppose therefore, that femur length and abdominal circumference will relate in the same direction to a single regulator; indeed, we have previously demonstrated differences in relationships between postnatal skeletal indices and femur length compared with abdominal circumference growth in utero [31]. These results support the notion that birth weight is a relatively crude surrogate for fetal developmental and that a more detailed measurement of individual markers of fetal growth may give a more accurate assessment of the regulation of development in utero.

A key question is what drives deregulated expression of *PHLDA2*? In rodent models *PHLDA2* responds to suboptimal in utero environments. Specifically, increased placental expression of *PHLDA2* has been reported in response to hypoxia during pregnancy, decreased food consumption and maternal alcohol consumption [37,38]. In this study, we noted that *PHLDA2* expression was higher in mothers who reported that they undertook strenuous exercise. A more extensive study will be critical in determining the relevance of this observation. Lower paternal birth weight was also associated with higher term placental *PHLDA2* mRNA levels. *PHLDA2* is imprinted and it is the paternally-inherited copy that is silenced. There is currently no evidence for full loss of imprinting of *PHLDA2* in low birth weight pregnancies [15,16] but increased expression could occur as a consequence of the failure of the paternal genome to fully silence *PHLDA2*. In which case, exploring the relationship between both maternal and paternal lifestyles will be important.

## Conclusion

In summary, higher expression of the placental growth regulator, *PHLDA2*, was associated with lower fetal femur growth velocity between 19 and 34 weeks gestation in fetuses who are within a normal birth weight range at birth. This suggests that the correct dosage of *PHLDA2* may be critical for optimal skeletal growth in the third trimester of pregnancy. Alterations in bone mineral content suggest that high placental *PHLDA2* may have long-term consequences for bone health. Different early life growth trajectories influence adult health and the identification of infants who have experienced sub-optimal growth using a molecular marker rather than by birth weight alone may be helpful in determining where to apply interventional strategies to improve long-term health.

The following are the supplementary materials related to this article.

**Supplementary figure:**
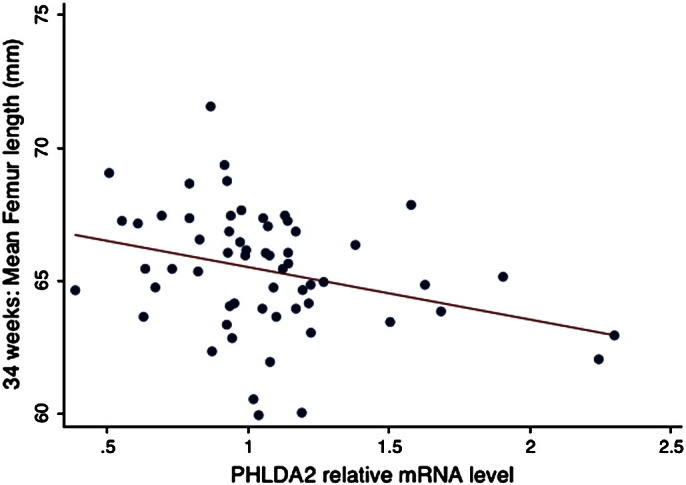
Scatter plot for absolute femur length at 34 weeks in mm. N = 58 subjects.

## Figures and Tables

**Table 1:** Pre-pregnant maternal and neonatal characteristics.

	Mean or median (SD or inter quartile range)	Number of subjects
Maternal age (years)	30.9 (3.9)	102
Maternal height (cm)	162.3 (6.5)	101
Maternal body mass index (kg/cm^2^)	25.2 (23.0–29.3)	101
Maternal mid upper arm circumference (cm)	29.8 (27.2–32.7)	101
Maternal arm muscle area (cm^2^)	36.7 (31.1–43.5)	101
Offspring's birth weight (g)	3503 (453)	102
Placental weight (g)	470 (96)	101
Birth weight/Placental weight ratio	7.5 (6.8–8.4)	101
Neonatal abdominal circumference (cm)	31.7 (1.8)	102
Neonatal crown heel length (cm)	49.7 (1.8)	102
Neonatal mid upper arm circumference (cm)	11.6 (1.0)	102

**Table 2:** Associations between placental *PHLDA2* expression and neonatal parameters. N = 102.

	*PHLDA2* relative mRNA levels	
	r	P
Birth weight	− 0.02	0.88
Placental weight	0.01	0.92
Placental/birth weight ratio	0.02	0.87
Head circumference	− 0.03	0.78
Abdominal circumference	0.03	0.76
Crown heel length	− 0.10	0.30
Ponderal index	0.09	0.36
Mid upper arm circumference	0.07	0.48
Neonatal bone mineral content	0.07	0.49
Neonatal lean mass	− 0.03	0.79
Neonatal fat mass	0.07	0.46

**Table 3:** Associations between placental *PHLDA2* expression and fetal parameters assessed by ultrasound. N = 59 at 19 weeks and 58 at 34 weeks. Significant correlations are highlighted in bold.

	*PHLDA2* relative mRNA levels	
	r	P
19 wk abdominal circumference z-score	0.01	0.93
34 wk abdominal circumference z-score	0.23	0.09
**19–34 wk abdominal circumference growth velocity z-score**	**0.28**	**0.04**
19 wk Head circumference z-score	0.15	0.27
34 wk head circumference z-score	0.005	0.97
19–34 wk head circumference growth velocity z-score	− 0.05	0.71
19 wk femur length z-score	0.13	0.34
34 wk femur length z-score	− 0.36	0.01
**19–34 wk femur length growth velocity z-score**	**− 0.42**	**0.001**

**Table 4:** Associations between placental *PHLDA2* expression and body composition assessed by DXA scan at age four years. N = 42. Significant correlations are highlighted in bold.

	*PHLDA2* relative mRNA levels	
	r	P
Bone mineral content	**− 0.38**	**0.01**
Bone area	**− 0.392**	**0.010**
Bone mineral density	**− 0.332**	**0.032**
Volumetric bone mineral density	− 0.147	0.353
Lean mass	− 0.23	0.15
Fat mass	− 0.20	0.21
4 year height	− 0.122	0.369
4 year weight (kg)	− 0.043	0.805
